# G Protein-Coupled Receptors in Osteoarthritis: A Novel Perspective on Pathogenesis and Treatment

**DOI:** 10.3389/fcell.2021.758220

**Published:** 2021-10-20

**Authors:** Ze-qin Wen, Di Liu, Yi Zhang, Zi-jun Cai, Wen-feng Xiao, Yu-sheng Li

**Affiliations:** ^1^Department of Orthopedics, Xiangya Hospital, Central South University, Changsha, China; ^2^Xiangya School of Medicine, Central South University, Changsha, China; ^3^National Clinical Research Center for Geriatric Disorders, Xiangya Hospital, Central South University, Changsha, China

**Keywords:** G protein-coupled receptor, osteoarthritis, cartilage matrix degradation, synovitis, pathogenesis, treatment

## Abstract

G protein-coupled receptors (GPCRs) are transmembrane receptor proteins that trigger numerous intracellular signaling pathways in response to the extracellular stimuli. The GPCRs superfamily contains enormous structural and functional diversity and mediates extensive biological processes. Until now, critical roles have been established in many diseases, including osteoarthritis (OA). Existing studies have shown that GPCRs play an important role in some OA-related pathogenesis, such as cartilage matrix degradation, synovitis, subchondral bone remodeling, and osteophyte formation. However, current pharmacological treatments are mostly symptomatic and there is a paucity of disease-modifying OA drugs so far. Targeting GPCRs is capable of inhibiting cartilage matrix degradation and synovitis and up-regulating cartilage matrix synthesis, providing a new therapeutic strategy for OA. In this review, we have comprehensively summarized the structures, biofunctions, and the novel roles of GPCRs in the pathogenesis and treatment of OA, which is expected to lay the foundation for the development of novel therapeutics against OA. Even though targeting GPCRs may ameliorate OA progression, many GPCRs-related therapeutic strategies are still in the pre-clinical stage and require further investigation.

## Introduction

Osteoarthritis (OA) is one of the most prevalent forms of arthritis and causes chronic pain, stiffness, swelling and loss of locomotion in the knees, hips, and hands (Xu et al., 2012). OA affects several joint structures and is characterized by articular cartilage degradation, subchondral bone sclerosis, osteophyte formation and synovial inflammation (Hunter and Bierma-Zeinstra, 2019). Age, obesity, sex, race, and genetics are considered the main risk factors for OA (Sharma, 2021). Drug intake, hospitalizations and joint surgeries related to the management of knee OA cost health care systems billions of dollars each year, which has caused a heavy socioeconomic burden (Dantas et al., 2021). However, current treatment modalities, including lifestyle changes, utilization of non-steroidal anti-inflammatory drugs (NSAIDs) and diacerein, and intra-articular injection of hyaluronic acid (HA), can only temporarily ameliorate local symptoms. Advanced OA patients inevitably have to undergo surgical interventions, such as artificial joint replacement (Wieland et al., 2005; Roos and Arden, 2016; Jones et al., 2019; Mlost et al., 2021). Therefore, OA is gradually becoming a global public health problem that requires further investigation.

G-protein-coupled receptors (GPCRs) are a family of more than 800 transmembrane proteins expressed in humans that regulate numerous physical processes, such as synaptic signaling, chemotaxis and metabolism (Wingler and Lefkowitz, 2020). The binding of extracellular ligands initiates the transduction of transmembrane signals by activating heterotrimeric G proteins, the phosphorylation of GPCRs, and the coupling of arrestin mediated by G-protein-coupled receptor kinases (GRKs) (Staus et al., 2016; Wang W. et al., 2018). Therefore, GPCRs are the most classic targets of two-thirds of existing therapeutic drugs used to treat a wide range of diseases, such as bone diseases, heart diseases, digestive diseases, and cancer (Kahsai et al., 2018; Nieto Gutierrez and McDonald, 2018; Wang J. et al., 2018; Gottesman-Katz et al., 2021).

In addition, it is worth noting that GPCRs play a critical role in the pathogenesis and treatment of OA. Destruction or mutation of GPCRs can lead to bone and joint dysfunction or diseases in humans, and most of these phenotypes have been validated in mouse models (Luo et al., 2019). Furthermore, emerging evidence has shown that GPCRs regulate the progression of OA by modulating cartilage matrix degradation, synovial inflammation, subchondral bone remodeling, osteophyte formation, chondrocyte hypertrophy, cartilage angiogenesis, and chondrocyte apoptosis (Jones et al., 2006; Yan et al., 2020; Mlost et al., 2021; Wang et al., 2021). However, the detailed mechanisms underlying the regulatory responses remain unclear. Therefore, this article will comprehensively review the novel roles of GPCRs in the pathogenesis and treatment of OA, aiming to explore the clinical application value of GPCRs.

## Novel Roles of G Protein-Coupled Receptors in Osteoarthritis

**FIGURE 1:**
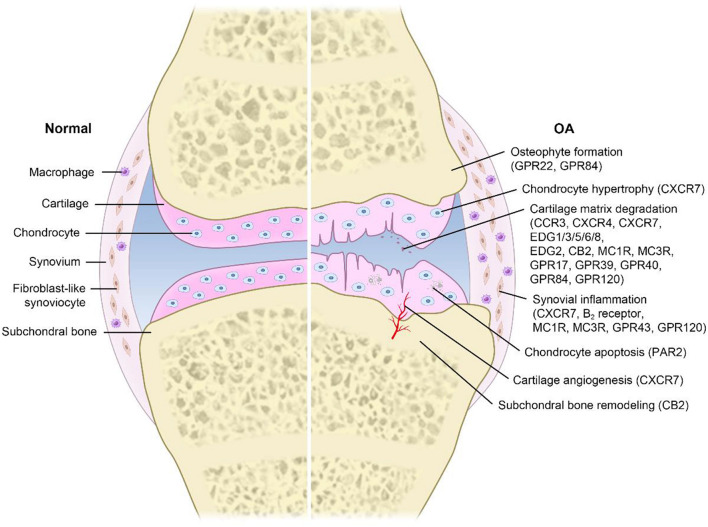
Pathogenesis in OA related to GPCRs. Different GPCRs are widely expressed on various cells and play a key role in transmembrane signal transmission. Extracellular stimuli initiate a series of intracellular signaling pathways by activating GPCRs, leading to a variety of physiological and pathological processes, such as cartilage matrix degradation, synovial inflammation, subchondral bone remodeling, osteophyte formation, chondrocyte hypertrophy, cartilage angiogenesis, and chondrocyte apoptosis. These processes greatly promote the occurrence and progression of OA.

Many studies have shown that targeting GPCRs can influence the pathogenesis and progression of OA (Neumann et al., 2014; Figure 1). However, the detailed mechanisms underlying the regulatory processes are still unclear. Moreover, the current treatment mainly relieves symptoms (it is unable to control the progression of the disease). A better understanding of the roles of GPCRs in OA is critical for developing a novel therapeutic strategy against OA. Therefore, we summarize known GPCRs that play important roles in OA (Table 1).

**TABLE 1:** Novel roles of GPCRs in OA.

GPCR family	GPCRs	Roles in pathogenesis of OA	Latent regulators	References
CKRs	CCR3	High concentrations inactivated cAMP/PKA and activated ERK and p38 MAPK, while at low concentrations activated PI3K and JNK MAPK to up-regulate MMP-3	U0126 SB203580	Chao et al., 2011
	CXCR4	Up-regulated the expression and release of MMP-3, MMP-9 and MMP-13, thus promoting the degradation and destruction of cartilage matrix	AMD3100	Yang et al., 2020
	CXCR7	Promoted chondrocyte hypertrophy, cartilage angiogenesis, cartilage matrix degradation, inflammation and endochondral ossification	NA	Jones et al., 2006
EDGs	EDG1/3/5/6/8	Increased PGE2 induced by COX-2 and MAPK to inhibit the expression of proteoglycan	NA	Masuko et al., 2007
	EDG2	Increased the expression of inflammatory cytokines and MMPs in synovial cells	NA	Mototani et al., 2008
CBs	CB2	Down-regulated MMP3 and MMP13 to improve subchondral bone morphology and underlying cartilage biochemical changes	HU308 WIN55,212-2	Mlost et al., 2021
PARs	PAR2	Inhibited apoptosis by activating P38/MAPK, NF-κB and PI3K/AKT/mTOR mediated autophagy in chondrocytes	AZ3451	Huang et al., 2019; Yan et al., 2020
Bradykinin receptors	B_2_ receptor	Led to pain and inflammation in the synovium	Icatibant MEN16132	Cucchi et al., 2005; Song et al., 2009
MCRs	MC1R	MC1R-deficient led to loss of collagen II and the increase of MMP-13 and pro-inflammatory cytokines and accelerated cartilage matrix changes	BMS-470539 C-terminal KPV	Lorenz et al., 2014
	MC3R	Inhibited the release of proinflammatory cytokines and MMPs	[DTrp8]-γ-MSH PG-990	Can et al., 2020
Secretin receptors	CTR	The expression of CTR in OA patients is significantly higher than that in normal controls	NA	Zupan et al., 2012

*CKRs, chemokine receptors; CCR, C-C chemokine receptor; CXCR, C-X-C chemokine receptor; EDGs, endothelial differentiation G-protein coupled receptors; CBs, cannabinoid receptors; PARs, protease activated receptors; MCRs, melanocortin receptors; MMP, matrix metalloproteinases; SNP, single nucleotide polymorphism; ECM, extracellular matrix; NA, not available.*

## Chemokine Receptors

There are two kinds of seven-helical molecules that bind chemokines: conventional chemokine receptors (cCKRs) and atypical chemokine receptors (ACKRs) (Hughes and Nibbs, 2018). cCKRs usually transduce signals through pertussis toxin-sensitive Ga_*i*_ G proteins and β-arrestins, eventually resulting in cell migration, adhesion, and other biological responses. Although four ACKRs are structurally related to cCKRs, they are not coupled to many signaling pathways activated by cCKRs.

CCR3 is a C-C chemokine receptor that functions by binding to its specific ligand eotaxin-1 (CXCL11) (Chang et al., 2016). Several studies have reported that increased eotaxin-1 secretion by chondrocytes and fibroblast-like synoviocytes (FLSs) can lead to the upregulation of matrix metalloproteinase 3 (MMP-3), matrix metalloproteinase 9 (MMP-9) and matrix metalloproteinase 13 (MMP-13) expression by binding to CCR3 but cannot induce eosinophil infiltration (Hsu et al., 2004; Neumann et al., 2014; Chang et al., 2016). In particular, high concentrations of eotaxin-1 can inhibit cAMP/PKA and activate ERK and p38 MAPK to regulate MMP expression, while at low concentrations, eotaxin-1 can activate PI3K and JNK MAPK to facilitate MMP secretion (Chao et al., 2011). Therefore, an ERK inhibitor (U0126) and p38 inhibitor (SB203580) can significantly reduce the expression of MMPs. The increased expression of MMPs plays a positive regulatory role in the progression of OA by promoting the degradation of cartilage matrix, suggesting that the eotaxin-1/CCR3 signaling pathway is a feasible target for treating OA.

CXCR4 is a C-X-C chemokine receptor that is related to the activation, differentiation and migration of immune cells by binding to the 8-kDa peptide stromal cell derived factor-1 (SDF-1/CXCL12) (Dong et al., 2016). A number of researchers have found a significant increase in SDF-1 concentrations in the synovial fluid of OA patients (Dong et al., 2016; Li et al., 2016). Moreover, the binding of SDF-1 and CXCR4 can upregulate the expression and release of MMP-3, MMP-9 and MMP-13, thus promoting the degradation and destruction of cartilage matrix (Yang et al., 2020). AMD3100, a class of bicyclams that influences HIV binding to normal cells, functions as a CXCR4 antagonist (Neumann et al., 2014; Dong et al., 2016). It can be used to inhibit the SDF-1/CXCR4 signaling pathway and protect chondrocytes and cartilage matrix from invasion. However, the expression levels of MMP-3, MMP-9 and MMP-13 were not reduced to normal levels by AMD3100 (Li et al., 2012). These results suggest that blocking the SDF-1/CXCR4 signaling pathway via AMD3100 is a possible treatment strategy.

CXCR7, also known as RDC1 and CCX-CKR2, formerly belonged to the class A orphan receptor GPCR and had certain homology with CKRs (Jones et al., 2006). CXCR7 was deorphanized and shown to be a CKR that binds to chemokines CXCL11 and CXCL12 (Miao et al., 2007). The activation of CXCR7 in cartilage tissue can promote cartilage matrix degradation, cartilage angiogenesis and chondrocyte hypertrophy, which facilitate the progression of OA. Furthermore, enhanced cartilage angiogenesis can result in a severe inflammatory response and endochondral ossification, driving chondrocytes to enter the early OA state (Jones et al., 2006). In addition to increased degradation of the cartilage matrix, the activation of CXCR7 reduces matrix synthesis and the production of the type 2A variant of type II collagen (Yang et al., 2015). Therefore, CXCR7 is a potential target to inhibit cartilage matrix degradation, cartilage angiogenesis, chondrocyte hypertrophy, and inflammation and improve chondral matrix synthesis in OA.

## Endothelial Differentiation G-Protein Coupled Receptors

The eight receptors of the EDG family can be activated by the phospholipid growth factors lysophosphatidic acid (LPA) and sphingosine-1-phosphate (S1P). The EDG family is divided into two groups based on their ligands. The S1P1/3/2/4/5 receptors (formerly EDG1/3/5/6/8) are specifically activated by S1P, while the LPA1/2/3 receptors (formerly EDG2/4/7) are specifically activated by LPA (Wang et al., 2001). The functions of EDGs vary, such as prolonging cell survival time, promoting cell proliferation and regulating deformability, adhesion, and chemotaxis (An et al., 1998; Wang et al., 2001).

EDG1/3/5/6/8, also known as S1P receptors, are GPCRs of the EDG family. S1P is a bioactive sphingolipid metabolite produced through phosphorylation of sphingosine. Sphingolipids are components of cell membranes and cellular signaling mediators, and almost all cells metabolize sphingolipids (Obinata and Hla, 2019). The S1P/EDG signaling pathway participates in a variety of cellular functions, such as proliferation, differentiation, migration, cytoskeletal rearrangement, adhesion, inflammation, and angiogenesis (Masuko et al., 2007; Obinata and Hla, 2019). Moreover, it has been reported that S1P receptors on human articular chondrocytes respond to S1P stimulation by significantly increasing the prostaglandin E2 (PGE2) production induced by COX-2 and MAPK, thereby inhibiting proteoglycan expression (Masuko et al., 2007). With the downregulation of proteoglycan expression, the cartilage matrix will not be able to renew normally and will therefore lose its original function and promote OA development.

Endothelial differentiation G-protein coupled receptor 2, also named the LPA1 receptor, is a GPCR of the EDG family. LPA can induce a variety of cellular responses in numerous types of cells, including proliferation and differentiation, morphological changes, chemotaxis, aggregation, and tissue invasion (Moolenaar et al., 1997). A stepwise association study reported that an SNP located in the promoter region of EDG2 was significantly associated with OA (Mototani et al., 2008). The LPA1 receptor encoded by EDG2 increases the expression of inflammatory cytokines and MMPs in synovial cells and may contribute to susceptibility to Japanese knee OA (Mototani et al., 2008).

## Cannabinoid Receptors

Cannabinoid receptors are the receptors of cannabinoid Δ9-tetrahydrocannabinol (THC), which is the bioactive component of marijuana. Currently, two major cannabinoid receptors have been identified, CB1 and CB2. CB1 receptors are mainly located in central and peripheral neurons, and their activation is primarily related to the downregulation of neuronal excitability, while CB2 receptors are mainly located in immune cells, and their activation is associated with reduced immune cell function, including decreased release of proinflammatory factors (Pertwee, 2008; Yang et al., 2015). CB1 is involved in mediating the psychoactivity of cannabis and the analgesic and antiemetic effects of THC, while CB2 plays a critical role in the pathophysiology of systemic inflammation, osteoporosis, central nervous system diseases and cancer (Atwood et al., 2012).

Cannabinoid receptor 2, a member of the GPCR family, responds to THC stimulation by modulating the inflammatory response (Howlett and Abood, 2017). Preclinical studies have revealed the important role of CB2 receptors in decreasing OA susceptibility, as the knockout of CB2 receptors leads to more serious cartilage degradation in surgical models of OA (Sophocleous et al., 2015). Long-term treatment with the CB2 selective agonist HU308 helps to relieve OA in the joint (Sophocleous et al., 2015). In addition, the mixed CB1 and CB2 agonists WIN55,212-2 have been shown to protect the cartilage matrix from degradation by reducing the expression of MMP-3 and MMP-13 in chondrocytes (Dunn et al., 2014). Compared with COX2 inhibitors, CB2 agonists can significantly reduce pain responses in OA patients, possibly because they offset central sensitization in OA patients at the molecular level (Mlost et al., 2021). CB2 agonists can improve subchondral bone morphology and underlying cartilage biochemical changes (Mlost et al., 2021). These results suggest that CB2 has great potential in the treatment and analgesia of OA. However, the existence of distinct differences between human and rat OA models must be taken into consideration. As a result, CB2 agonists should be studied in animal models, which are closer to the actual situation in humans, to verify their therapeutic effect in treating human OA.

## Protease-Activated Receptors

Protease-activated receptors (PARs) are important members of the GPCR family that are activated by serine proteases, such as thrombin, trypsin, and MMPs (Elste and Petersen, 2010; Neumann et al., 2014). PARs have been divided into four subtypes (PAR1–PAR4). In contrast to canonical receptors, PARs can be activated by N-terminal proteolytic cleavage. The resulting N-terminal peptides without a particular peptide act as tethered activation ligands, interacting with the ECL2 domain, and initiating downstream signaling (Heuberger and Schuepbach, 2019). In the classical signaling pathway, activated receptors transduce signals by recruiting G proteins. However, the alternative activation of PARs can induce the transactivation and signal transduction of receptors, including colocalized PAR (Heuberger and Schuepbach, 2019).

Protease-activated receptor-2 is a critical factor affecting the pathogenesis of several diseases, such as inflammatory, gastrointestinal, respiratory and metabolic diseases (Yau et al., 2016). Activation of PAR-2 may stimulate the secretion of the inflammatory cytokines IL-1β, IL-6, and IL-8 in peripheral blood mononuclear cells (Johansson et al., 2005). Furthermore, researchers have observed that the expression of PAR-2 in OA chondrocytes is markedly upregulated compared to that in normal chondrocytes (Xiang et al., 2006). Similarly, PAR-2-deficient (PAR2^–^/^–^) mice have been reported to be conspicuously protected against cartilage damage and osteosclerosis in an OA model caused by destabilization of the medial meniscus (DMM) (Huesa et al., 2016). The results above suggest that PAR-2 plays a vital role in the occurrence and progression of OA. Therefore, the PAR-2 antagonist AZ3451 inhibits chondrocyte apoptosis to improve OA by activating chondrocyte autophagy by regulating the P38/MAPK, NF-κB, and PI3K/AKT/mTOR signaling pathways (Huang et al., 2019; Yan et al., 2020).

## Bradykinin Receptors

Two bradykinin receptor subtypes, B_1_ receptor and B_2_ receptor, have been identified and are classified as Class I GPCRs (IUPHARs) (De Falco et al., 2013; Neumann et al., 2014). B_1_ receptors mediate the action of C-terminal desArg metabolites, while B_2_ receptors mediate the action of bradykinin (BK) and Lys-BK (De Falco et al., 2013). The +9/−9 polymorphism of the B_2_ receptor (BDKRB_2_ +9/−9 polymorphism) has been reported to be a genetic marker for the pathogenesis and development of OA (De Falco et al., 2013). BK is formed in plasma and inflammatory tissues and initiates several processes, including vasodilation, plasma extravasation, immune system activation and chemotaxis induction of leukocytes by activating B_2_ receptors present in the membranes of various cell types (Meini and Maggi, 2008). BK in particular has a great effect on the occurrence of pain and the inflammatory response.

The B_2_ receptor can trigger a signaling cascade that leads to pain and inflammatory effects in the synovium when activated (Neumann et al., 2014). B_2_ receptors have been identified on synovial lining cells, fibroblasts, and endothelial lining cells in the vessels of patients with OA, while there is no evidence to support the existence of B_1_ receptors (Meini and Maggi, 2008). In addition, icatibant is a synthetic decapeptide and antagonist of the B_2_ receptor that is currently used for angioedema attacks. A clinical study reported that icatibant was effective in reducing pain intensity in patients with OA, and its analgesic activity was more significant during activity than at rest (De Falco et al., 2013). However, no anti-inflammatory effect has been observed (Song et al., 2009). MEN16132 is a novel potent and selective B_2_ receptor antagonist that is also known as fasitibant (Cucchi et al., 2005). It can block inflammatory responses in human synovial fibroblasts, especially the BK-induced release of IL-6 and IL-8 (Neumann et al., 2014). A clinical study called ALBATROSS confirmed the effects of MEN16132 in humans (De Falco et al., 2013).

## Melanocortin Receptors

Melanocortin receptors are receptors of proopiomelanocortin (POMC) and its derived peptides, and five MCR subtypes, MC1R-MC5R, have been cloned thus far (Renquist et al., 2011; Lorenz et al., 2014). POMC is a versatile precursor protein for a variety of hormones, including melanocyte-stimulating hormones (α-MSH, β-MSH, and γ-MSH) and adrenocorticotropic hormone (ACTH) (Lowry, 2016; Wang et al., 2019). POMC is involved in a variety of biological processes, such as the maintenance of energy metabolism balance, nociceptive sensation and the regulation of exocrine gland function and the immune system (Wang et al., 2019). Although original neurohormones were induced by stress in the classic hypothalamic-pituitary-adrenal (HPA) axis, it has now been shown that POMC and its derived peptides can also be generated autonomously in many peripheral tissues, such as skin and joints (Lorenz et al., 2014).MC1R is a member of the GPCR family. The transcripts of MC1R, MC2R, and MC5R have been shown to be present in articular chondrocytes derived from patients with OA. A study reported that the activation of MC1R leads to antiarthritic effects by inducing synovial tissue aging and cartilage protection *in vivo* (Montero-Melendez et al., 2020). In contrast, another study found that MC1R signal-deficient mice showed an OA-related cartilage phenotype prior to OA induction, suggesting an early stage of OA (Lorenz et al., 2014). Specifically, a lack of MC1R signaling facilitates age-related cartilage matrix changes, such as loss of collagen II and an increase in the number of MMP-13-positive chondrocytes (Lorenz et al., 2014). Given the important role of MC1R in OA, MC1R agonists such as BMS-470539 dihydrochloride and C-terminal KPV can delay the progression of OA. Moreover, it was observed that the MC3R agonists [DTrp^8^]-γ-MSH and PG-990 inhibited the release of proinflammatory cytokines and MMPs to a greater extent than the MC1R agonist when administered prophylactically and therapeutically, suggesting greater potential than MC1R (Can et al., 2020). Therefore, activation of MC1R and MC3R may be effective therapeutic strategies against OA.

## Calcitonin Receptor

Calcitonin receptor, also known as CALCR, is one of the oldest members of the class B GPCR family. CTR has been considered a common therapeutic target for osteoporosis, as CTR is involved in the regulation of bone loss and osteoclast survival (Lee et al., 2020). Moreover, a study observed that the expression of CTR in OA patients was obviously higher than that in normal controls (Zupan et al., 2012). However, another previous controlled study of OA patients and cadavers found no difference in CTR expression (Kuliwaba et al., 2000). Therefore, whether there are differences in the expression of CTR between OA patients and normal controls and the role of CTR in the pathogenesis of OA remain to be researched.

## Other 7TM Receptors

Several GPCRs do not belong to any family of the GRAFS classification system. Therefore, these receptors are named by other 7TM receptors. Most of them belong to orphan receptors of GPCRs. Seven GPCRs relevant to OA belong to other 7TM receptors (Table 2).

**TABLE 2:** Novel roles of other 7TM receptors in OA.

GPCRs	Roles in pathogenesis of OA	Latent regulators	References
GPR17	Down-regulated the expression of MMP-3 and MMP-13, thereby inhibited the degradation of type II collagen	Pranlukast	Wang et al., 2020
GPR22	Contained an SNP associated with OA	NA	Kerkhof et al., 2010
GPR39	Down-regulated the expression of MMP-3, MMP-13 and ADAMTS to reduce the degradation of type II collagen and aggrecan and reversed the decrease of TIMP-1 and TIMP-2 expression	TC-G1008 AGEs	Shan et al., 2019
GPR40	Down-regulated the expression of MMP-3 and MMP-13 to inhibite the degradation of type II collagen and suppressed the activation of NF-κB signaling pathway	GW9508	Gu et al., 2020
GPR43	Reduced the release of pro-inflammatory mediators and adhesion molecules, inhibiting inflammatory signaling pathways	Butyrate	Pirozzi et al., 2018
GPR84	Modulated the expression of MMPs and ECM synthesis to regulate the pathogenesis of OA	6-OAU Lauric acid	Wang et al., 2021
GPR120	Down-regulated the expression of IL-6 and IL-8 and protected type II collagen and aggrecan by reversing the decrease in SOX9 expression	NA	Xu et al., 2020

*NA, not available.*

GPR17 is a GPCR coupled to the Gi subunit and is also an orphan receptor, primarily confined to the oligodendrocyte lineage, which is critical for the timing of oligodendrocyte myelination (Ou et al., 2019; Wang et al., 2020). Due to the wide distribution of GPR17 in the CNS, it is often considered a classic target for brain diseases, including multiple sclerosis (MS) and neuronal damage (Zhao et al., 2018; Nyamoya et al., 2019). The structure of GPR17 is phylogenetically related to P2Y and cysteinyl-leukotriene (CysLT) receptors and consists of seven transmembrane domains connected by loops (Saravanan et al., 2018; Wang et al., 2020). Moreover, it has been reported that MDL29951, T0510-3657 and AC1MLNKK are possible ligands of GPR17 (Eberini et al., 2011; Hennen et al., 2013; Saravanan et al., 2018).

Tumor necrosis factor α (TNF-α) is one of the most pivotal proinflammatory cytokines in the progression of OA (Zhao et al., 2019). TNF-α triggers a series of responses through the JAK2/STAT1/IRF-1 signaling pathway to upregulate the expression of MMP-3 and MMP-13, thereby promoting the degradation of type II collagen (Richardson and Dodge, 2000; Xu et al., 2018). Pranlukast is a leukotriene receptor antagonist (LTRA) used as a therapeutic drug in asthma patients (Trinh et al., 2019). Moreover, pranlukast has been considered a synthetic inhibitor of GPR17 (Wang et al., 2020). It has been demonstrated that pranlukast has protective effects on TNF-α-induced degradation of type II collagen by blocking GPR17 expression, which suggests that targeting GPR17 may be a possible therapeutic strategy for OA.

GPR22 is also an orphan receptor. A recent genome-wide association scan (GWAS) of Dutch Caucasian OA patients found a locus on GPR22 that was related to knee and/or hand OA (Raine et al., 2012). Similarly, a study found the existence of GPR22 in cartilage and osteophytes of mouse OA models, while it was absent in normal cartilage (Kerkhof et al., 2010). Therefore, these results suggest that GPR22 is engaged in the pathogenesis of OA.

GPR39 is a conserved protein expressed in vertebrates and is associated with insulin secretion, synaptic signaling, gastric emptying, and depression (Zhao et al., 2015). Formerly considered an orphan receptor, zinc ions were later identified as endogenous agonists of GPR39 and are potential targets for selective zinc ion regulation (Holst et al., 2007; Lu et al., 2019; Shan et al., 2019). TC-G 1008, chemically known as 2-pyridine pyridine, was originally developed to improve GLP-1 levels in people with type 2 diabetes. Currently, TC-G 1008 has been confirmed to activate GPR39 and hence alleviate IL-1β-induced chondrocyte senescence, showing a protective effect on chondrocytes (Lu et al., 2019). In addition, the activation of GPR39 can downregulate the expression of MMP-3, MMP-13, and a disintegrin and metalloproteinase with thrombospondin motifs (ADAMTS), reduce the degradation of type II collagen and aggrecan and reverse the decrease in TIMP-1 and TIMP-2 expression (Shan et al., 2019). However, the expression of GPR39 in SW1353 chondrocytes is inhibited by contact with advanced glycation end products (AGEs), promoting the progression of OA. Therefore, GPR39 plays an important role in OA, and targeted activation of GPR39 can inhibit the progression of OA.

GPR40, a long-chain fatty acid receptor, is the most highly expressed GPCR in islet β cells and is also abundantly expressed in intestinal L cells (Syed et al., 2018). It can promote the release of GLP-1 together with GPR120. In addition, GPR40 is also expressed in leukocytes, macrophages and bone marrow stromal cells, which share a common precursor with the bone and cartilage lineages (Monfoulet et al., 2015; Philippe et al., 2017). It has been shown that GPR40 knockout (GPR40^–^/^–^) mice exhibit symptoms of osteoporosis, while activation of GPR40 improves bone mineral density (Wauquier et al., 2013). Even so, the lack of GPR40 alone is insufficient to induce significant histological changes in cartilage or changes in basal chondrocyte metabolism related to OA (Monfoulet et al., 2015). However, the characteristics of induced OA were much more serious in GPR40-deficient models, suggesting that GPR40 activation could alleviate or slow the progression of OA. GW9508, the selective agonist of GPR40, could significantly downregulate the expression of MMP-3 and MMP-13 to inhibit the degradation of type II collagen against the stimulation of AGEs and suppress the activation of the NF-κB signaling pathway, showing a protective effect on OA (Gu et al., 2020).

GPR43, a GPCR commonly existing in human adipocytes, colonic epithelial cells and peripheral blood mononuclear cells, can be activated by short-chain fatty acids (SCFAs) (Ang and Ding, 2016). SCFAs such as acetate (C2), propionate (C3), and butyrate (C4) are generated by gut bacteria during the fermentation of dietary fiber. Butyrate can regulate inflammatory diseases both inside and outside the intestine through GPR43. In addition, binding between GPR43 and butyrate has been shown to be effective against acute arthritis by inhibiting the expression of proinflammatory mediators, adhesion factors, and MMPs and maintaining the homeostasis of bone metabolism (Young et al., 2005; Chabane et al., 2008; Canani et al., 2011; Pirozzi et al., 2018). However, there is little evidence of its therapeutic effect on OA. Therefore, further research on the roles of GPR43 in the pathogenesis of OA is needed.

GPR84, a member of the metabolic GPCR family, is a medium-chain fatty acid (MCFA) receptor that can be specifically activated by C9-C12 saturated fatty acids. It was first identified in 2001, binds to the toxin-sensitive Gα_*i*_ protein of *Bordetella pertussis* and inhibits adenylate cyclase activity (Wang et al., 2006; Nicol et al., 2015). GPR84 is not a formal “deorphanized” receptor because whether MCFAs are the primary endogenous ligands that activate is controversial GPR84 (Mahmud et al., 2017). GPR84 is primarily expressed in immune cells and is involved in the inflammatory response, but its mechanism of modulating inflammation has not been fully described (Recio et al., 2018). In addition, GPR84^–^/^–^ mice exhibit increased catabolism and decreased anabolism, significantly aggravating articular cartilage degradation, osteophyte development, and subchondral bone remodeling; these results prove that GPR84 is involved in the pathogenesis of OA in mice (Wang et al., 2021). In contrast, the GPR84 agonist 6-OAU or lauric acid could protect human OA cartilage explants by upregulating the expression of genes related to cartilage anabolic metabolism. Therefore, GPR84 is a therapeutic target with great potential.

GPR120, also known as free fatty acid receptor 4 (FFAR4), is the receptor of ω-3 fatty acids. It is widely distributed in various tissues and cells, such as intestinal tissue, adipose tissue, macrophages, and pancreas, and performs a wide range of physiological functions, such as regulating the secretion of gut hormones and insulin (Oh et al., 2010; Mo et al., 2013; Ichimura et al., 2014). The main components of fish oil, ω-3 FA (docosahexaenoic acid (C22:6N3 and DHA) and eicosapentaenoic acid (C20:5N3 and EPA), can produce potent anti-inflammatory effects through GPR120 (Oh et al., 2010). Moreover, activation of GPR120 can inhibit inflammation by downregulating IL-1β-induced expression of IL-6 and IL-8 and protect type II collagen and aggrecan against degradation by reversing the decrease in SOX9 expression (Chen et al., 2018; Xu et al., 2020). In general, GPR120 is involved in the pathogenesis of OA by controlling the inflammatory response, metabolic homeostasis, and osteoclast differentiation. Therefore, the increase in miR-15b-5p caused by the downregulation of LINC00662 is able to downregulate the expression of GPR120, thereby promoting the progression of OA (Lu and Zhou, 2020). In conclusion, some receptors of the GPCR family have a critical effect on the occurrence and progression of OA by regulating the destruction of the cartilage matrix, subchondral bone remodeling, inflammation, and chondrocyte autophagy. We can delay the progression and alleviate the symptoms of OA to some extent by targeting these important GPCRs. However, many of these therapeutic strategies are still in the preclinical stage, and whether they are effective in patients with OA remains unknown. Given the key role of GPCRs in OA, it is significant to explore the specific mechanism by which GPCRs influence OA in order to facilitate the early diagnosis and treatment of OA.

## Conclusion

G protein-coupled receptors are ubiquitously expressed seven-transmembrane-domain receptors and mediate the transduction of transmembrane signals. Activated GPCRs induce a series of downstream signaling cascades and subsequent pathophysiological responses by interacting with G proteins, GRKs, and arrestin. GPCRs are involved in the occurrence and progression of OA by regulating some pathological processes, such as cartilage matrix degradation, synovitis, subchondral bone remodeling, and osteophyte formation. Most importantly, GPCRs play a key role in cartilage matrix degradation and synovial inflammation. Current evidence has demonstrated that GPCRs can enhance the expression of MMPs (e.g., MMP-3, MMP-9, and MMP-13), ADAMTS and proinflammatory cytokines (e.g., IL-1β, IL-6, IL-8, and TNF-α) and promote cartilage matrix degradation and synovial inflammation in OA. Moreover, targeting GPCRs principally by inhibiting cartilage matrix degradation and synovial inflammation and by upregulating cartilage matrix synthesis could mitigate OA. However, most of the current GPCR-related therapeutic strategies are still at early stages, and the safety and efficiency of *in vivo* experiments remain unknown. Further studies are still warranted to further explore the issues discussed in this review.

## Author Contributions

Z-QW, DL, YZ, and Z-JC decided on the content, wrote the manuscript, and prepared the figures. W-FX and Y-SL conceived and revised this review. All authors approved the final version of the manuscript and agreed to be accountable for all specs of the work.

## Conflict of Interest

The authors declare that the research was conducted in the absence of any commercial or financial relationships that could be construed as a potential conflict of interest.

## Publisher’s Note

All claims expressed in this article are solely those of the authors and do not necessarily represent those of their affiliated organizations, or those of the publisher, the editors and the reviewers. Any product that may be evaluated in this article, or claim that may be made by its manufacturer, is not guaranteed or endorsed by the publisher.
